# Volatile and Nonvolatile Contributors to Lager Beer Acceptability in High-Flavor-Liking Consumers

**DOI:** 10.1021/acs.jafc.5c16579

**Published:** 2026-04-28

**Authors:** Joel Borcherding, Megan Booth, Said Toro Uribe, Diana Paola Forero, Edisson Tello, Julie Peterson, Devin G. Peterson

**Affiliations:** Department of Food Science and Technology, Pelotonia Research Center, 2647 The Ohio State University, 2255 Kenny Rd, Columbus, Ohio 43210, United States

**Keywords:** untargeted flavoromics approach, gas chromatography-olfactometry strategies, chemical profiling, consumer acceptability, lager beer, high-flavor-liking consumers

## Abstract

Untargeted flavoromics and complementary analytical techniques were integrated to identify chemical drivers of consumer liking among individuals who prefer high-flavor-intensity lager beers. Untargeted liquid chromatography–mass spectrometry and gas chromatography–mass spectrometry profiling enabled global characterization of volatile and nonvolatile constituents across samples, which were modeled against flavor-liking scores using multivariate analysis. Odorants detected by gas chromatography-olfactometry were further characterized by targeted tandem mass spectrometry and incorporated into the chemometric framework. Compounds exhibiting the highest predictive scores were identified, revealing 11 positively and four negatively correlated with flavor liking. Sensory recombination experiments confirmed that these compounds significantly influenced flavor perception and overall liking, validating their contribution to consumer preference. Notably, four nonvolatile compounds (feruloyl 3-hydroxagmatine, 2′-deoxyadenosine, *p*-coumaroyl 3-hydroxyagmatine, and N^1^,N^10^-diferuloylspermidine) were not perceived in water by a sensory panel, yet influenced flavor perception and disliking in the beer sample. These findings advance the molecular understanding of flavor liking in beer.

## Graphical abstract

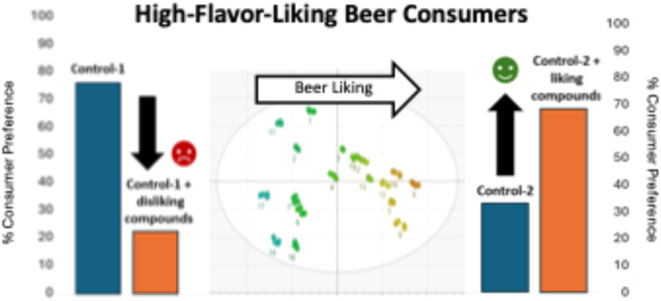

## Introduction

Beer holds both cultural and economic significance as the world’s most widely consumed alcoholic beverage, with a market that consists of both traditional styles and the rapidly expanding craft movement. Although produced from four core ingredients, beer contains a diverse array of compounds that result in a broad range of flavors. Differences in starting materials, brewing methods, and fermentation practices yield distinct flavor profiles to cater to a diverse range of consumer preferences.^1–5^

Recent research has focused on enhancing the understanding of consumer preferences, particularly among the growing demographic of consumers who identify as “craft beer drinkers,” who tend to seek out bold, distinctive flavors.^6^ Historically, macro-breweries have focused on producing mild-flavored lagers; however, in response to shifting market dynamics, they are now creating “craft-style” beers to capitalize on the demand for more flavor-forward offerings.^7^ Given the global dominance of lagers in the beer market over other styles, there is considerable commercial interest in identifying key flavor drivers that contribute to consumer liking within a demographic that prefers high-flavor intensity beers.^8^ Recent studies have utilized predictive models to identify compounds that influence the complex flavor of beer and consumer liking. However, these studies have primarily focused on volatile aroma compounds, examining their effects on overall consumer preference without considering how demographic differences shape liking.^9–11^ In the initial phase of this study, the authors identified both volatile and nonvolatile compounds that impact consumer preference, specifically among consumers who prefer low-flavor lagers, using a combination of targeted chemical profiling of detected aroma compounds and untargeted flavoromics techniques.^12^ Flavoromics enables the integration of chemical profiling data with sensory outcomes, such as consumer liking, through multivariate analysis. A key advantage of combining targeted chemical profiling of detected odorants with untargeted analytical approaches is the enhanced identification of trace compounds that may otherwise be missed by untargeted methods alone. This integrated strategy, which melds untargeted flavoromics with targeted chemical profiling of compounds identified by gas chromatography–olfactometry (GC‑O), facilitates a more focused and quantitative evaluation of selected compounds. Together, these approaches enable a more comprehensive characterization of the beer samples by identifying subthreshold and suprathreshold flavor compounds and modulators that contribute to the sensory profile and drive consumer liking.^13^

In the second phase of this two part study, the flavor liking of lager beers among high-flavor-liking consumers was investigated, integrating comprehensive chemical profiling with untargeted liquid chromatography/mass spectrometry (LC/MS) and gas chromatography/mass spectrometry (GC/MS) and targeted gas chromatography–tandem mass spectrometry (GC/MS/MS) approaches across 18 lager beer samples to enhance the understanding between chemical composition and consumer preference.

## Materials and Methods

### Beer Samples and Chemicals

Eighteen lager beers were sourced from a commercial retailer (Columbus, OH) and maintained at 4 °C until sensory evaluation.^12^ The beers were chosen to represent a variety of lager styles with differing chemical and flavor profiles, while ensuring ethanol content remained comparable (4.4–5.2%). For each brand, beers were from the same lot and within their manufacturer’s stated best-by date. Nanopure water (18.2 MΩ) was produced using a Barnstead GenPure Pro system (Thermo Fisher Scientific, Waltham, MA). Food-grade ethanol (200 proof), Optima LC/MS grade acetonitrile, formic acid, methanol, acetone, anhydrous sodium sulfate, and 2-deoxyadenosine (≥99% pure) were purchased from Thermo Fisher Scientific (Waltham, MA). Diethyl ether (≥99% pure), and all GC/MS standards indicated in Table S1 (≥99% pure) were purchased from Sigma-Aldrich (St. Louis, MO) (Supporting Information). Pyroglutamyl leucine (≥95% pure) was purchased from A2B Chem (San Diego, CA). Synthetic standards for feruloyl 3-hydroxyagmatine (≥95% pure), *p*-Coumaroyl 3-hydroxyagmatine (≥95%), and N^1^,N^10^-diferuloylspermidine (≥95% pure) were purchased from WuXi AppTec (Shanghai, China). Nonvolatile standards were further purified by SPE and verified by LC/MS.

### Consumer Acceptance Test and Cluster Analysis

One-hundred-thirty-four (71 female, 63 male, ages 21–65+) consumer panelists participated in an acceptance test evaluating 18 lager beers. The consumer test and cluster analysis were carried out as previously described.^12^ In brief, over three sessions, panelists evaluated six beers (1.5 oz) at refrigerated temperature (4 °C). Samples were randomized and counterbalanced across sessions and labeled with randomized three-digit codes. Samples were prepared just before serving to minimize effects from changes in carbonation and temperature. Aroma, color, flavor, and overall liking were assessed using a standard nine-point hedonic liking scale (1 = dislike extremely, 5 = neither like nor dislike, and 9 = like extremely). Just-about-right (JAR) questions were used to evaluate the intensity of aroma, color, flavor, bitterness, sweetness, and carbonation in the beer samples using a five-point scale (1 = much too little, 2 = slightly too little, 3 = just about right, 4 = slightly too strong, and 5 = much too strong). Water was used as a palate cleanser between samples. Data was collected on Compusense Cloud Software (Compusense, Inc., Guelph, ON, Canada). The study was approved by the Ohio State Internal Review Board (IRB) protocol #2023B0023.

Consumers were segmented based on flavor-liking scores using hierarchical clustering on principal components (HCPC) with Ward’s linkage method and Euclidean distances,^14^ as previously described.^12^ The number of clusters was selected based on inspection of the hierarchical dendrogram, which showed an initial and robust split into two main groups. This two-cluster solution was retained to maintain statistical power and interpretability and was defined as low-flavor-liking and high-flavor-liking consumer segments. Liking scores were analyzed by two-way analysis of variance (ANOVA) followed by Tukey’s HSD posthoc analysis (α = 0.05) and Spearman correlation analysis. The high-flavor-liking consumers were further characterized based on JAR questions, demographic information, and beer consumption habits.

### Untargeted Chemical Profiling by LC/MS-QToF

Untargeted nonvolatile chemical profiles of the beer samples were collected using sample preparation, LC/MS analysis, and data processing as previously described.^12^ In brief, beer samples were prepared in biological quadruplicates (different cans/bottles). Each sample underwent freeze-drying, reconstitution, homogenization, and centrifugation. Before injection, supernatants of each sample were diluted 4-fold with nanopure water and filtered through a polytetrafluoroethylene filter (PTFE, 0.45 μm, Sigma-Aldrich, St. Louis, MO). A quality control (QC) sample was prepared by pooling equal volume aliquots of each diluted beer sample.

The analysis was carried out using an Agilent 1290 Infinity II UPLC system coupled to an Agilent 6546 Q-ToF Mass Spectrometer (Agilent Technologies, Santa Clara, CA) with a Jet Stream Electrospray Ionization Source (AJS ESI). Samples (1 μL) were injected onto a Cortecs T3 (2.1 × 150 mm, 1.6 μm, Waters Corp., Milford, MA) at a flow rate of 0.5 mL/min using acidified water (0.1% formic acid) and acidified acetonitrile (0.1% formic acid) as solvents A and B, respectively. Elution gradient was 0–2 min, 0% B; 2–17 min, 100% B; 17–19.5 min, holding 100% B. Mass spectrometric analysis was conducted using electrospray ionization (ESI) in both positive and negative modes, with a scan range of 100–1700 Da and a rate of two spectra per second.

LC/MS chemical profiling data were processed in MassHunter Profinder version 10.0 (Agilent Technologies, Wood Dale, IL) for retention time alignment and feature extraction. Extracted features and corresponding peak areas were exported to R for data processing. Features detected in blanks or exhibiting coefficients of variation (CV) exceeding 20% across QC replicate injections were excluded from further analysis. Nonvolatile compounds were identified through comprehensive library searches (HBMD, FooDB, and Metlin) and subsequently confirmed by MS/MS fragmentation patterns, accurate mass measurements, and retention time alignment with authentic standards.

### Untargeted Chemical Profiling by GC/MS-QToF

The untargeted chemical profiling of the volatile compounds in the beer samples was collected using sample preparation, GC/MS analysis, and data processing as previously described.^12^ In brief, beer samples were degassed and extracted using both an immersive thin film solid phase microextraction (TF-SPME) and stir bar sorptive extraction (SBSE) methods (4 replicates). Chemical profiles were collected using an Agilent 7890B GC (Agilent Technologies, Santa Clara, CA) coupled to an Agilent 7250 Q-ToF mass spectrometer (Agilent Technologies, Santa Clara, CA) and equipped with a Gerstel MPS robotics autosampler (MPS2, Gerstel, Mülheim an der Ruhr, Germany). Compounds were separated on a DB-WAX Ultra Inert capillary column (60 m × 0.25 mm × 0.25 μm, Agilent Technologies, Santa Clara, CA) with a programmed oven temperature gradient. Mass spectrometry operated in electron ionization (EI) mode (70 eV) with a scan range of 35–350 *m*/*z*. Sample order was randomized, and blank and quality control samples were included throughout to ensure analytical reliability. Compounds were identified by comparison with authentic standards using linear retention indexes in two columns (DB-Wax and DB-5), database matching (NIST 17), and spectral information (MS, MS/MS).

GC/MS chemical profiling data were initially deconvoluted using MassHunter Unknowns Analysis v10.0 (Agilent Technologies, Santa Clara, CA) with the SureMass algorithm. Feature alignment was subsequently performed in Mass Profiler Professional v14.9.1 (Agilent Technologies, Santa Clara, CA), and the aligned data was exported to MassHunter Quantitative Analysis for recursive feature extraction and internal standard normalization. Further data processing in R involved the removal of features detected in blank samples and those exhibiting a coefficient of variation (CV) greater than 30% across the QC replicate injections.

### GC/MS-O Analysis and Targeted Chemical Profiling by GC/MS/MS

Aroma compounds were identified in a pooled QC sample using gas chromatography/mass spectrometry-olfactometry (GC/MS-O) as described previously.^12^ In brief, sample preparation was carried out using two different extraction methods: immersive thin-film solid-phase microextraction with stir bar sorptive extraction (TF-SPME-SBSE), and solvent extraction with diethyl ether followed by solvent-assisted flavor evaporation (SAFE).^15^ For solvent extraction, the QC sample (300 mL) was spiked with an internal standard (2-methyl-3-heptanone, 10 μL, 1000 mg/L in ethanol), and gently shaken, dried with anhydrous sodium sulfate and subjected to vacuum distillation using a SAFE apparatus at 40 °C followed by solvent removal in a Vigreux column until a final extract volume of 1 mL and stored at −80 °C for further analysis. GC-O analysis was conducted by two experienced panelists, with each extract (SAFE and TF-SPME-SBSE) evaluated in duplicate using two chromatographic columns (DB-5MS, DB-Wax). Compounds detected in at least two of four GC-O sessions were classified as aroma compounds. The GC/MS-O system consisted of an Agilent 7890B GC coupled to an Agilent 5977 mass spectrometer (Agilent Technologies, Santa Clara, CA), and equipped with a DB-Wax or DB–5MS capillary columns (both 60 m x 0.25 mm x 0.25 μm, Agilent Technologies, Santa Clara, CA), sniffing port (ODP, Gerstel, Mülheim an der Ruhr, Germany), and a MPS autosampler (Gerstel, Mülheim an der Ruhr, Germany). Identification of aroma compounds was based on linear retention indices (LRI) from both DB-5 and DB-Wax columns, supported by mass spectral data and odor descriptors, and verified using authentic standards.

Identified aroma compounds were further profiled across all the beer samples in triplicate using targeted gas chromatography-tandem mass spectrometry (GC/MS/MS) as previously described.^12^ Two TF-SPME-SBSE-GC/MS/MS methods were developed based on compounds’ abundance. All analyses were conducted on an Agilent 7890*B*/7010B triple quadrupole GC/MS system equipped with a DB-Wax column (60 m × 0.32 mm × 0.25 μm, Agilent Technologies, Santa Clara, CA) with a programmed oven temperature gradient. The mass spectrometer was operated in EI mode (70 eV), and multiple reaction monitoring (MRM) transitions for each odor-active compound were optimized according to the abundance and selectivity of the precursor-to-product ion transition using authentic standards (Supporting Information).

### Multivariate Statistical Analysis

Chemical profiling and sensory data were exported and analyzed in SIMCA (v17, Sartorius, Germany) using principal component analysis (PCA) for exploratory pattern recognition and orthogonal partial least-squares (OPLS) regression to model relationships between chemical composition and consumer liking. Both LC/MS and GC/MS–based data sets were log-transformed prior to multivariate analysis. LC/MS data were Pareto-scaled, while GC/MS and GC–MS/MS data were scaled to unit variance. In the OPLS models, chemical profiling was used as *x*-variables, and the consumer liking scores were used as *y*-variables to identify relationships between chemical composition and sensory preference. Model validation employed 6-fold cross-validation and 100-permutation testing, and top-ranked variables were selected based on VIP predictive scores and loadings correlations (p­(corr)) as previously described.^12^

### Identification and Quantitation of Predictive Compounds of Beer Liking

#### Volatile Compounds

Volatile compounds were identified using linear retention indexes on two columns (DB-Wax and DB-5), database matching (NIST 17), and spectral information (MS, MS/MS) by comparison with authentic standards, corresponding to level 1 identification confidence. Full compound-level evidence is available in the Supporting Information of our previous publication.^12^ The quantitation of the predictive volatile compounds (Table 1) was performed using an Agilent 8860 GC (Agilent Technologies, Santa Clara, CA) coupled to an Agilent 7010C Triple Quadrupole MS system (Agilent Technologies, Santa Clara, CA), and equipped with a Gerstel MPS autosampler (Gerstel, Mülheim an der Ruhr, Germany). Degassed beer (5 mL) was diluted with nanopure water (5 mL) in a 10 mL screw-capped vial. Either ethanol or a mixture of target compounds in ethanol (10 μL) was added, followed by the addition of an internal standard (10 μL, 10 mg/L 2-methyl-3-heptanone in ethanol). Sample extraction using TF-SPME-SBSE and analysis was performed under the same conditions described above for the Targeted GC/MS-O and GC/MS/MS Analysis. The MS was operated in EI mode (70 eV) using MRM optimized transitions (Supporting Information). Quantitation was carried out using a seven-point standard addition method in which authentic commercial standards were introduced at multiple concentrations within the sample matrix. Calibration curves were built in triplicate and displayed good linearity for all the compounds (*R* ^2^ ≥ 0.989) (Supporting Information).

**Table 1:** Top Ranked Nonvolatile (top) and Volatile (bottom) Variables of Importance (VIP) Sorted by Correlation to Modeled Flavor Liking (p­(corr)) in the OPLS Predictive Component

								concentration in beer samples^f^	
	**#**	data set^a^	compound ID	VIPpred^b^	p(corr)^c^	sensory attribute^d^	detection threshold^e^	most liked (#1)	least liked (#18)
nonvolatile	1	ESI(−)	Pyroglutamyl leucine	5.7	0.59	rich, sour^27^	2	7.5 ± 0.42	4.2 ± 0.39
	2	ESI(+)	Feruloyl 3-hydroxagmatine	6.8	–0.55	NR	NR	1.2 ± 0.05	2.8 ± 0.14
	3	ESI(+)	2′-Deoxyadenosine	6.7	–0.71	NR	NR	5.9 ± 0.38	32.6 ± 1.6
	4	ESI(+)	*p*-coumaroyl 3-hydroxyagmatine	5.9	–0.55	NR	NR	2.6 ± 0.13	5.4 ± 0.53
	5	ESI(+)	N1,N10-diferuloylspermidine	5.5	–0.66	NR	NR	0.04 ± 0.004	0.07 ± 0.002
volatile	6	U	2-Acetylpyrrole	2.1	0.71	musty, nutty, sweet^46^	58,600	694 ± 14	65 ± 14
	7	U & T	Ethyl 2-methylpropanoate	2.0	0.68	Fruity^(i)^	0.1	9.55 ± 1.11	0.96 ± 0.01
	8	U & T	Dihydromaltol	1.9	0.67	Caramel^(i)^	50	5653 ± 448	513 ± 32
	9	U & T	4-Hydroxy-2,5-dimethyl-3(2H)-furanone	1.9	0.66	Caramel^(i)^	300	2353 ± 354	327 ± 27
	10	U	2-Acetylfuran	1.9	0.65	Balsamic, sweet^46^	10,000	39.9 ± 2.5	6.3 ± 1.2
	11	U	Ethyl tiglate	1.9	0.64	Fruity^46^	63	1.15 ± 0.08	0.07 ± 0.01
	12	U & T	Ethyl 3-methylbutanoate	1.8	0.62	Fruity, berry^(i)^	0.01	4.07 ± 0.17	0.23 ± 0.02
	13	U & T	Geraniol	1.8	0.61	Floral, rose^(i)^	141	194.6 ± 1.8	3.11 ± 0.19
	14	U & T	Ethyl 2-methylbutanoate	1.7	0.57	Fruity, berry^(i)^	0.006	1.78 ± 0.21	0.13 ± 0.03
	15	U	Ethyl 3-methylthiopropionate	1.6	0.55	Sulfuric, fruity^46^	8.45	1.76 ± 0.05	0.23 ± 0.02

a U stands for untargeted and T stands for targeted.

b Cut-off values were >5.5 for nonvolatiles and ≥ 1.5 for volatiles. Scores are from N models in cases for compounds selected in both N & T.

c Cut-off values were ≥ |0.55|.

d Reported aroma according to indicated sources or ^(i)^GC/O analysis of the QC beer samples. NR stands for not reported.

e Orthonasal thresholds in water for volatiles, taste threshold in sake for nonvolatiles. Units are mg/L for nonvolatiles, μg/L for volatiles. NR stands for not reported. Threshold values reported in sources indicated in sensory attribute column.

f Mean ± standard deviation from biological triplicates. Units are mg/L for nonvolatiles, μg/L for volatiles.

#### Nonvolatile Compounds

Nonvolatile predictive compounds (Table 1) were annotated through library searches (HMDB, FooDB, Metlin) and confirmed by MS/MS fragmentation, accurate mass, and retention time relative to commercial or synthetic standards, corresponding to level 1 identification confidence. Quantitation was performed using a 5-point standard addition method with authentic commercial standards added at multilevels within the sample matrix. Degassed beer samples (#1 and #18, Figure 1) were diluted 100-fold with water for the quantitation of all predictive compounds. A mixture of the selected compounds in water was added using a standard addition protocol, followed by an internal standard (propyl paraben, 1.5 mg/L, 100 μL). The final volume of each sample was then adjusted to 800 μL prior to injection.

**Figure 1:**
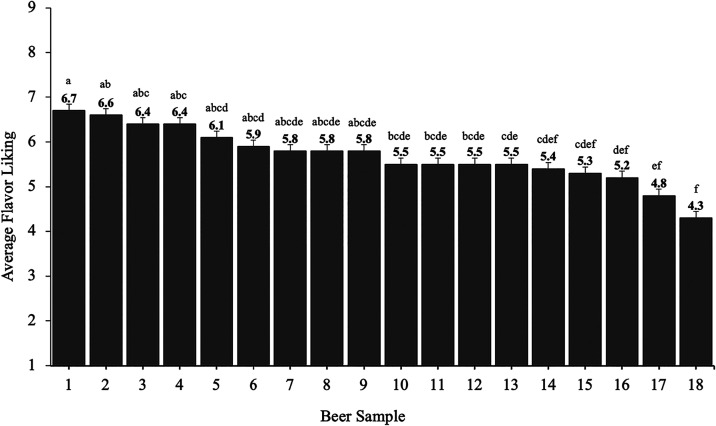
Average flavor liking scores of the beer samples for high-flavor-liking consumers cluster (*n* = 66) on a 9-point hedonic scale. Different letters indicate significant differences in liking (*p* < 0.05) according to Tukey’s HSD test; error bars represent standard errors.

Analysis was performed using a Waters Acquity UPLC H-Class system coupled to a Xevo TQ-XS mass spectrometer (Waters Co., Milford, MA) and equipped with a Cortecs C18 column (2.1 × 100 mm × 1.6 μm, Waters Co., Milford, MA). Samples (1 μL) were injected with a constant flow rate of 0.5 mL/min using a binary solvent gradient of A (nanopure water with 0.1% formic acid) and B (acetonitrile with 0.1% formic acid). The compounds were separated via the following gradient: 0–1 min, 0% B; 1–7 min, 100% B; 7–10 min, holding 100% B; 10–10.1 min, 0% B; 10.1–12 min, 100% B. Column was re-equilibrated for 2 min at initial conditions between injections. Mass spectrometer parameters were as follows: capillary voltage: 2.2 kV; desolvation temperature: 600 °C, desolvation flow rate: 1000 L/h, cone gas flow rate: 150 L/min, source temperature: 150 °C. The mass spectrometer was operated in ESI (+) mode using multiple reaction monitoring (MRM) (Supporting Information). Standard addition calibration curves with 3 biological replicates demonstrated good linearity for all the compounds (*R* ^2^ ≥ 0.99) (Supporting Information).

### Sensory Validation

The sensory impact of the predictive compounds of flavor liking was validated through two approaches: difference testing and consumer evaluation. For all validation testing, the least liked (sample #18, Figure 1) and most liked (sample #1, Figure 1) beers were used as sample matrices. To prepare both the recombination and control samples, beer was weighed (100 g) into a frozen beaker to minimize volume variations related to foaming. For the recombination samples, compounds solubilized in ethanol were spiked into the respective beer samples immediately before sensory evaluation to match the concentrations quantitated in the least and most liked samples. An equivalent volume of ethanol was added to the control samples to account for the slightly altered ethanol content of the spiked sample. Samples were placed in beakers and then stirred with a spatula to uniformly distribute the compounds (verified by instrumental analysis). Samples (20 mL) were pipetted into clear 2.5 oz glasses (2.5 oz) labeled with 3-digit blinding codes for the evaluation.

#### Difference Testing

The sensory impact of the predictive compounds was evaluated using triangle testing. Eighteen panelists (7 male, 11 female, ages 22–46) were recruited internally from the Flavor Research and Education Center at The Ohio State University and followed the protocol previously described.^12^ In brief, panelists participated in a series of triangle tests in which they were instructed to taste three samples, identify the one that differed from the other two, and then comment on the perceived difference. The sample order within each set was randomized, and each set was evaluated in duplicate. Significant differences were determined by performing two-sided binomial tests (chance probability of 1/3).

To evaluate if the positively correlated volatile compounds altered flavor perception two recombination models (models A and B) were prepared (Table 2), grouping the compounds based on their concentrations relative to reported sensory thresholds: Model A (compounds above threshold values): ethyl 2-methylpropanoate (7.94 μg/L), dihydromaltol (5160 μg/L), 4-hydroxy-2,5-dimethyl-3­(2H)-furanone (2008 μg/L), ethyl 3-methylbutanoate (3.59 μg/L), geraniol (190.0 μg/L), and ethyl 2-methylbutanoate (1.61 μg/L); Model B (compounds below threshold values): 2-acetylpyrrole (631.0 μg/L), 2-acetylfuran (34.0 μg/L), ethyl tiglate (1.11 μg/L), and ethyl 3-methylthiopropionate (1.48 μg/L). To prepare each recombination model, a mixture of the respective volatile aroma compounds in ethanol (50 μL) was added to 100 g of the least-liked beer (sample 18, Figure 1), with an equivalent volume of pure ethanol (50 μL) added to the control (sample 18).

**Table 2:** Recombination Models to Validate the Sensory Activity of Predictive Positively and Negatively Correlated Compounds of Beer Liking by Triangle Difference Testing

		concentration added to recombination samples (μg/L)			
predictive compound	correlation to beer liking	Model A^a^	Model B^b^	Model C^c^	Model D^d^
2-Acetylpyrrole	+		631		
Ethyl 2-methylpropanoate	+	7.9			
Dihydromaltol	+	5160			
4-Hydroxy-2,5-dimethyl-3(2H)-furanone	+	2008			
2-Acetylfuran	+		34.0		
Ethyl tiglate	+		1.1		
Ethyl 3-methylbutanoate	+	3.6			
Geraniol	+	190			
Ethyl 2-methylbutanoate	+	1.6			
Ethyl 3-methylthiopropionate	+		1.5		
Feruloyl 3-hydroxyagmatine	–			2800	1600
2-Deoxyadenosine	–			32600	26700
*p*-Coumaroyl 3-hydroxyagmatine	–			5400	2800
N^1^, N^10^-Diferuloylspermidine	–			70.0	30.0

a (+) Positively correlated compounds with concentrations detected above their odor threshold values added to least liked sample (#18) to mimic the concentrations of most liked sample (#1).

b (+) Positively correlated compounds with concentrations detected below their odor threshold values added to least liked sample (#18) to mimic the concentrations of most liked sample (#1).

c (–) Negatively correlated compounds added to water that mimic the concentrations in least liked sample (#18).

d (–) Negatively correlated compounds added to most liked sample (#1) to mimic the concentration in least liked sample (#18).

To evaluate the sensory impact of the four negatively correlated nonvolatile predictive compounds, two recombination samples were prepared (models C and D) in beer and in water, respectively (Table 2). Since these compounds lack reported flavor activity or sensory thresholds, the water matrix was included to determine whether they possess inherent flavor properties: Model C (water matrix): feruloyl 3-hydroxyagmatine (2800 μg/L), 2-deoxyadenosine (32,600.0 μg/L), *p*-coumaroyl 3-hydroxyagmatine (5400 μg/L), and N^1^,N^10^-diferuloylspermidine (70.0 μg/L); Model D (beer matrix): feruloyl 3-hydroxyagmatine (1600.0 μg/L), 2-deoxyadenosine (26700 μg/L), *p*-coumaroyl 3-hydroxyagmatine (2800 μg/L), and N^1^,N^10^-diferuloylspermidine (30 μg/L) The least-liked beer (sample 18, Figure 1) was the basis for the concentrations of the 4 compounds evaluated in Models C and D. A mixture of the four negatively correlated compounds in ethanol (50 μL) was added to prepare the recombination samples in the control beer (sample 1) and water (100 g), with equivalent volumes of ethanol added to the controls (50 μL). To ensure uniform distribution of the spiked compounds, aliquots were taken from three different locations within each solution and analyzed by LC to confirm homogeneous mixing. See Table 2 for the composition of recombination samples. The study was approved by the Ohio State Internal Review Board (IRB) protocol #2023B0406.

#### Consumer Paired Preference Testing

A paired preference test was conducted to assess the impact of the positively and negatively correlated compounds on consumer liking and disliking, respectively. Lager beer consumers who participated in the original acceptance test were recruited, and 32 of the 66 consumers who were segmented into the high-flavor liking cluster participated. Two recombination models were prepared by the addition of either positively or negatively correlated compounds to their respective control beer samples (Table 3). To prepare a recombination model with compounds that were predictive of liking, the positively correlated above and below threshold volatile aroma compounds previously found to significantly influence beer flavor perception (triangle tests), as well as the nonvolatile compound pyroglutamyl leucine, were added to the control sample (least liked beer, sample #18, Figure 1) at the levels reported the most liked beer (sample #1, Figure 1). To prepare a recombination model with the compounds that were predictive of disliking, the 4 negatively correlated nonvolatile compounds previously found to significantly influence beer flavor perception (triangle tests), were added to the most liked control beer (sample #1, Figure 1) at the levels in the least-liked beer (sample #18, Figure 1), see Table 3.

**Table 3:** Recombination Models to Validate the Sensory Activity of Predictive Compounds by Consumer Preference Testing

predictive compound	correlation to beer liking	concentration added to recombination beer sample (μg/L)^a^
2-Acetylpyrrole	+	629
Ethyl 2-methylpropanoate	+	8.59
Dihydromaltol	+	5140
4-Hydroxy-2,5-dimethyl-3(2H)-furanone	+	2026
2-Acetylfuran	+	33.6
Ethyl tiglate	+	1.08
Ethyl 3-methylbutanoate	+	3.84
Geraniol	+	191.49
Ethyl 2-methylbutanoate	+	1.65
Ethyl 3-methylthiopropionate	+	1.53
Pyroglutamyl leucine	+	3270
Feruloyl 3-hydroxyagmatine	–	1600
2-deoxyadenosine	–	26700
*p*-coumaroyl 3-hydroxyagmatine	–	2800
N^1^,N^10^-diferuloylspermidine	–	30

a (+) Positively correlated compounds added to least-liked sample (#18) to mimic concentrations of most liked sample (#1); (–) Negatively correlated compounds added to most-liked sample (#1) to mimic concentrations of least-liked sample (#18).

Consumers were presented with two sample sets in successive order, each consisting of a recombination model and its respective control. Consumers tasted each sample and selected their preferred beer, with the option of making a comment explaining their choice. Subsequently, they rated flavor liking for each sample on a 9-point hedonic scale. Preference and liking differences were analyzed using a one-sided binomial test (chance level = 0.5) and a one-sided paired *t* test, respectively. One-sided analyses were performed with the expectation that positively and negatively correlated samples would increase and decrease liking scores of beer samples, respectively. Testing was conducted in climate-controlled sensory booths under ambient lighting, and water was used as a palate cleanser. All data were collected on Compusense Cloud Software (Compusense Inc., Guelph, ON, Canada). The study was approved by the Ohio State Internal Review Board (IRB) protocol #2024B0132.

## Results and Discussion

### Acceptability of Lager Beer Flavor Across Different Consumer Segments

Tello et al. reported that cluster dendrogram analysis of flavor-liking score distributions for 18 lager beers identified two distinct consumer segments: those preferring beers with lower flavor and those favoring higher flavored beers.^12^ PCA was first applied to the flavor liking data, and five principal components explaining 54.2% of the variance were retained prior to clustering. Consumers were segmented based on flavor liking scores using HCPC with Euclidean distances and Ward’s linkage method. Inspection of the hierarchical dendrogram revealed an initial and robust split into two consumer groups of comparable size (n = 66 and 68), defined as high-flavor-liking and low-flavor-liking consumers, a solution that preserved interpretability and statistical power. In the present study, the high-flavor-liking group was further examined as part of this two-phase investigation to identify chemical drivers of liking. Overall liking was strongly correlated with flavor liking (Spearman’s ρ = 0.92), whereas aroma liking (ρ = 0.45) and color liking (ρ = 0.45) were comparatively weak-to-moderately correlated with overall liking. Penalty analysis of the demographic data identified “too strong bitterness”, “too weak sweetness”, and “too weak flavor” as top drivers of disliking for high-flavor-liking consumers (Figure 2). These results reinforce prior findings that flavor attributes, rather than visual appearance or aroma alone, are the primary drivers of consumer acceptance in beer.^16^

**Figure 2:**
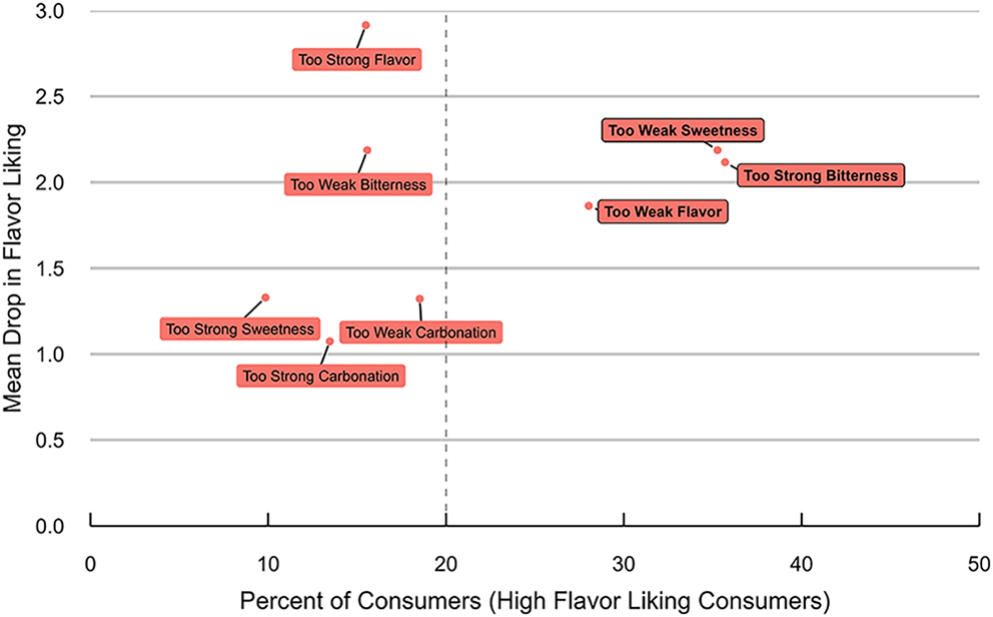
Mean drops in flavor liking for high-flavor-liking consumers as a function of the percentage of consumers and appropriateness of bitterness, sweetness, flavor, and carbonation intensity across all beer samples; reproduced from Tello et al.^12^ Copyright [2025] American Chemical Society.

The average flavor liking scores for all 18 beer brands for high-flavor-liking consumers are reported in Figure 1. The liking scores ranged from 4.3 (dislike slightly) to 6.7 (like moderately) and were highest for beers classified in the amber lager style family (samples #1–3), which are known for having a malty flavor with caramel accents and a balanced, restrained bitterness.^17^ These results support high-flavor-liking consumers’ preferences toward lager beers with stronger flavor profiles and lower bitterness.

### Chemical Profiling of Beer Samples

The composition of 18 lager beers was characterized using both untargeted and targeted chemical profiling approaches. Nonvolatile compounds were profiled across all samples by LC/MS in both ESI positive and negative modes, yielding 2196 and 2381 chemical features, respectively. Volatile compounds were analyzed using untargeted GC/MS, resulting in the detection of 181 features. Additionally, 38 odor compounds were previously identified from a blend of all the beers by GC/MS-O analysis.^12^ Techniques such as GC/MS-O aid in the identification of key aroma molecules that directly contribute to the flavor profile of a beer. Complementing the targeted approach, untargeted profiling provides a neutral chemical profile independent of sensorial activity, enabling the discovery of modulators that may lack direct sensory attributes at the concentrations reported. Together, these analytical strategies yield distinct yet complementary insights, contributing to a more comprehensive understanding of the beer flavor chemistry.

### Multivariate Analysis and Selection of Predictive Compounds of Beer Liking

Principal component analysis (PCA) was initially applied to the chemical profiles of the 18 lager beers, spanning LC/MS (ESI+ and ESI-) and both targeted and untargeted GC/MS data sets, to visualize sample variation. Score plots showed tight clustering of biological replicates, confirming strong analytical reproducibility. However, beer samples did not separate clearly by style (pale, amber, pilsner), likely due to diverse ingredient compositions (e.g., hop and barley varieties, adjuncts, yeast strains) and processing conditions (e.g., mashing, fermentation, maturation).

Orthogonal partial least-squares (OPLS) regression models were subsequently developed to predict consumer beer liking based on chemical profiling data. Nonvolatile compounds from LC/MS and volatile compounds from GC/MS were used as *X*-variables, and the average flavor liking scores from high-flavor-liking consumers were used as the *Y*-variable.^18^ The resulting score plots (Figures 3 and 4) illustrate clear sample separation along the first component, corresponding to differences in flavor preference. In addition, all models demonstrated high predictive power for explaining the variation in flavor liking (*R* ^2^ *Y* ≥ 0.95, *Q* ^2^ ≥ 0.93, RMSE_cv_ ≤ 0.39), with the risk of overfitting minimized according to *p*-values from analysis of variance (ANOVA) of the cross-validated residuals and permutation test intercepts.^19,20^ Subsequently, chemical features with top-ranked variable of importance in prediction scores (VIPpred >1.5 for GC/MS, VIPpred >5.5 for LC/MS) and correlations in the OPLS predictive first component (p­(corr) ≥ 0.55 for positively correlated compounds, p­(corr) ≤ −0.55 for negatively correlated compounds) were selected for further investigation (Table 1).^21,22^

**Figure 3:**
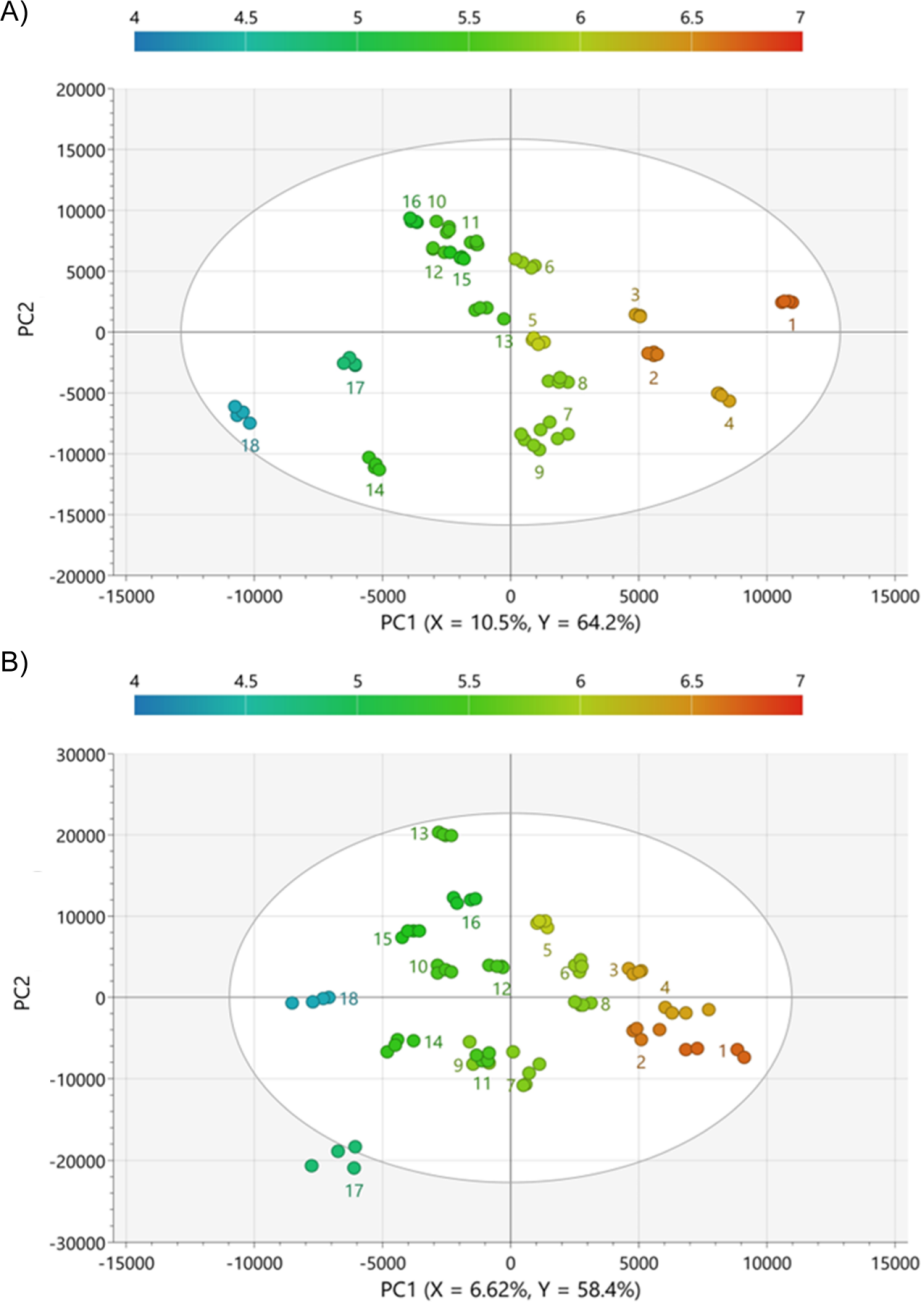
Scores plots from the OPLS models relating the LC/MS profiles of the 18 lager beers to the flavor liking scores of (A) ESI­(+) data and (B) ESI(−) data. Samples are colored according to the liking scores.

**Figure 4:**
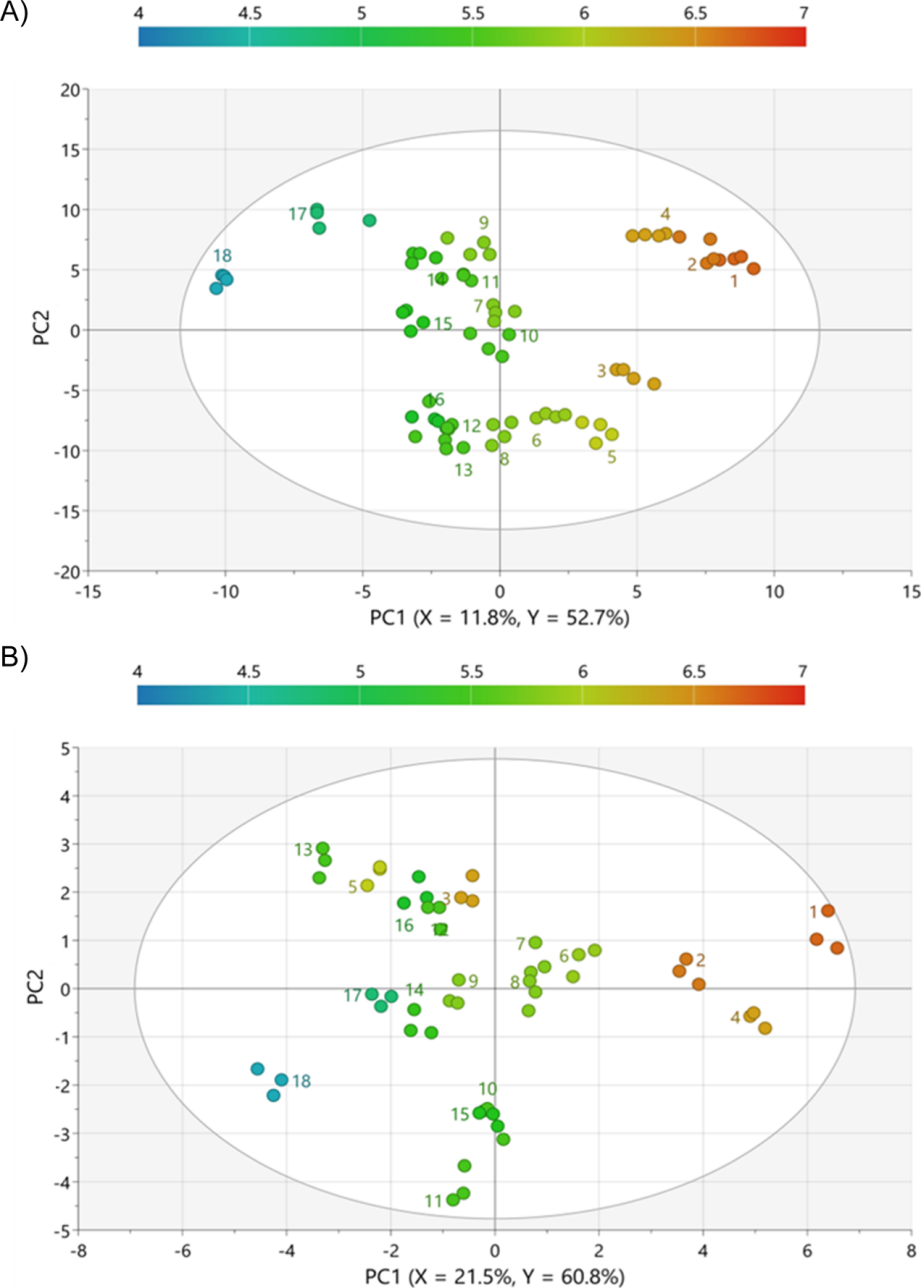
Scores plots from the OPLS models relating the GC/MS profiles of the 18 lager beers to the flavor liking scores: (A) untargeted compounds and (B) targeted aroma compounds. Samples are colored according to the liking scores.

### Identification of Predictive Compounds

Among the top predictors of flavor liking, 11 compounds (10 volatile and 1 nonvolatile) showed positive correlations, while 4 nonvolatile compounds were negatively correlated. Six of the positively correlated compounds, identified via targeted GC/MS-O, included ethyl 2-methylpropanoate (fruity), ethyl 3-methylbutanoate (fruity, berry-like), ethyl 2-methylbutanoate (fruity, berry-like), dihydromaltol (caramel), 4-hydroxy-2,5-dimethyl-3­(2H)-furanone (caramel), and geraniol (floral). The concentration of these compounds is influenced by factors such as fermentation conditions, yeast strain, malt type, and hop variety.^23–26^ Conversely, 4 volatile compounds (2-acetylpyrrole, 2-acetylfuran, ethyl tiglate, and ethyl 3-methylthiopropionate) were identified exclusively through untargeted GC/MS analysis, and although they were not identified as aroma compounds by GC/MS/O analysis, they were found to be strong predictors of consumers’ preference among high-flavor-liking beer drinkers, suggesting subthreshold activity. Previous work on volatiles highly correlated to bottom-fermented beer liking identified other compounds, such as 4-ethylguaiacol and trans-2-ionone.^11^ While not determined to be highly predictive of lager beer liking in the present study, the importance of other notable beer compounds should not be discounted. The highly predictive compounds identified in this model may only account for beer liking within the high-flavor-liking demographic or across the 18 beers analyzed. Notably, only one nonvolatile compound was positively correlated to liking, identified as pyroglutamyl leucine, an amino acid previously characterized as imparting rich and sour taste in sake.^27^

On the other hand, all the negatively correlated compounds were nonvolatile and identified as feruloyl 3-hydroxagmatine, 2′-deoxyadenosine, *p*-coumaroyl 3-hydroxyagmatine, and N^1^,N^10^-diferuloylspermidine based on their accurate mass (ESI±), MS/MS fragmentation patterns, and confirmed with synthetic or authentic commercial standards. The agmatine and spermidine analogues have previously been identified in barley, beer, sorghum, and rice, with their purported role being in defense against insects.^28–31^ Analogues of spermidine have previously been described as the primary bitter component in lulo fruit pulp, which may explain their negative correlation to consumer liking in the present study.^32^

The compounds that drive consumer liking can vary between beer styles (e.g., ale vs lager) due to differences in personal preferences and consumer expectations for a given product category.^33^ However, the present findings extend beyond cross-style comparisons to demonstrate that variation in consumer preferences also emerges within a single style (i.e., lager). This intrastyle variation was characterized by predictive volatile and nonvolatile compounds that are above and below sensory thresholds. Consumer liking of lager beer was therefore shaped by chemical composition and individual preference, rather than a monolithic response to the category as a whole.

### Quantitation of Predictive Compounds of Beer Liking

To assess differences in concentration among key predictors of flavor liking and to establish a foundation for recombination models in sensory validation, the predictive volatile and nonvolatile compounds were quantitated in the most and least liked beer samples (samples #1 and #18, respectively). The observed patterns were consistent with the modeled correlations: compounds showing positive correlations with liking were present at higher levels in the most-liked beer, whereas negatively correlated compounds were more abundant in the least-liked beer (Table 1).

Among the compounds positively correlated with flavor liking, pyroglutamyl leucine was the only nonvolatile and was determined to be 1.8-fold higher in the most liked beer compared to the least liked beer. Notably, this peptide has also been detected at similar concentrations in sake matrices, highlighting its potential sensory relevance.^27^ In contrast, the volatile compounds showed even more pronounced differences, ranging from 6.3 to 62.6-fold higher in the most liked beer, with six of the 10 exceeding their sensory threshold values. As expected, these above-threshold volatiles were consistently identified in the targeted analysis, underscoring their contribution to perceived flavor liking. It is important to note that the threshold values reported in Table 1 were not determined in a beer matrix. As a result, discrepancies may exist between the reported thresholds in water and those applicable to the analyzed beer. However, thresholds measured in water are typically lower than those in ethanol-containing matrices,^34^ meaning that water-based thresholds may overestimate aroma activity in beer. Several of the predictive volatile compounds, including 2-acetylpyrrole, dihydromaltol, 4-hydroxy-2,5-dimethyl-3­(2H)-furanone, 2-acetylfuran, and 2′-deoxyadenosine, are derived from malt.^26,35,36^ This is in alignment with the style of the most liked beer, a dry-hopped, amber-style lager (sample #1, Figure 1), which is known for being malt-forward. Conversely, the least liked beer was a pale-style lager (sample #18, Figure 1), which has been characterized by a more neutral flavor profile.^17^ Collectively, these findings suggest that the preferences of high-flavor-liking consumers are partially driven by a greater abundance of aroma-active volatiles, particularly those associated with malt character.

On the other hand, the four nonvolatile compounds that were negatively correlated with liking, feruloyl 3-hydroxagmatine, 2′-deoxyadenosine, *p*-coumaroyl 3-hydroxyagmatine, and N^1^,N^10^-diferuloylspermidine, were quantitated at levels 1.8 to 5.5-fold higher in the least liked beer compared to the most-liked beer. While spermidine analogues have been reported as contributing to bitter taste, these agmatines and spermidines had no previously reported sensory attributes, suggesting a previously unrecognized contribution to beer flavor.^32^

In order to validate the impact of the positively and negatively correlated compounds, the concentrations of these compounds in fresh beer samples were subsequently adjusted to match quantitated levels in the most or least liked beers and subjected to sensory evaluation to discern causal relationships between flavor liking and the predictive compounds.

### Sensory Validation

#### Sensory Difference Testing of Predictive Compounds

Difference testing revealed that the highly predictive compounds listed in Table 1 contributed to perceptible differences between the most and least liked beer samples. While it is commonly assumed that only compounds quantitated at concentrations above detection thresholds have aroma impact, previous studies have demonstrated that below-threshold compounds can modulate perception in alcoholic beverages, including beer and wine.^37–39^ Therefore, the impact of concentration differences between the most and least liked beer samples for the above and below threshold groups of positively correlated compounds were evaluated separately. The addition of the positively correlated above threshold compounds resulted in significant perceptual differences (*p* = 0.002), confirming their flavor contribution. Panelists commented that the recombination sample had a stronger fruity, floral, hoppy flavor and less bitter taste than the control, which aligned with the compounds’ positive correlation to liking and results from the penalty analysis (Figure 2). The addition of positively correlated below-threshold volatile compounds also indicated perceptible changes, though at a lower significance level (*p* = 0.03). Panelists commented that the recombination sample was milder and less bitter than the control. The significant impact of the below-threshold compounds indicates that receptor modulation, together with cross-modal olfactory–gustatory interactions, may alter neural integration in ways that suppress off-flavors and bitterness.^40–42^ The sensory activity for the 4 highly predictive nonvolatile compounds without existing threshold data was evaluated via difference testing in both water and beer matrices. No significant perceptual differences were detected in the water matrix (*p* = 0.4), indicating that these compounds were not direct contributors to flavor perception. In contrast, when evaluated in the most liked beer (sample 1), the compounds induced a significant perceptual difference (*p* = 0.009) with panelists commenting that the recombination beer was more bitter, irritating, and sharper than the control. These findings suggest that the negatively correlated nonvolatile compounds modulate flavor perception through interactions within the beer matrix, rather than through their individual taste properties. Previous research has reported that the presence of alcohol can impact the sensory attributes of phenolic compounds. For instance, the addition of phenolic compounds to low-alcohol wine increased the perception of bitterness and heat.^43^ Furthermore, agmatines are known precursors of hordatines, which are secondary metabolites of barley and can be generated during the fermentation and malting process.^31,44^ These hordatines have been described as contributing to astringency in beer aftertaste, which may suggest similar activity from agmatine precursors.^45^

#### Consumer Paired Preference Validation

A paired preference test was conducted to evaluate the influence of positively and negatively correlated compounds on consumer liking and disliking in lager beer. Two recombination models were prepared by adding either positively or negatively correlated compounds to their respective control beer samples (Table 3). To assess the impact of the positively correlated volatile aroma compounds previously found to significantly influence beer flavor perception (triangle tests), as well as the positively correlated nonvolatile compound pyroglutamyl leucine, were added to the least-liked beer (sample #18, Figure 1) at the levels reported in the most liked beer (sample #1, Figure 1). Pyroglutamyl leucine was previously reported to impart a rich and sour taste to sake at concentrations above 2 mg/L, and it was quantitated above this concentration in both the most- and least-liked beers (Table 1)^27^ The majority of consumers (67%) significantly preferred the recombination beer containing the positively correlated compounds over the control (*p* = 0.035), with liking scores of 5.7 and 5.5, respectively. Panelists who preferred the recombination sample commented that the beer was crisper, smoother, and sweeter, and that it had more flavor and aroma. Because the positively correlated compounds were added to a previously disliked beer, the small change in liking score was not unexpected, as the underlying negative attributes of the beer were still present. However, in the preference test, consumers consistently favored the recombination over the control, demonstrating that even in a previously disliked beer, these positively correlated compounds can meaningfully shift preference among high-flavor-liking consumers.

A larger effect size was observed for the four negatively correlated predictive nonvolatile compounds: feruloyl 3-hydroxyagmatine, 2-deoxyadenosine, *p*-coumaroyl 3-hydroxyagmatine, and N^1^,N^10^-diferuloylspermidine. When these four compounds were spiked into the most liked control beer (sample #1, Figure 1) to match the levels found in the least liked beer (sample #18, Figure 1), 77% consumers significantly preferred the control (*p* = 0.002), rating their liking of the samples as 6.6 and 5.3, respectively. Consumers commented that the flavor of the recombination sample was more bitter with a harsh aftertaste. These results indicate that, although these nonvolatile compounds showed no sensory activity in water, they acted as flavor-modulators in beer, reducing consumer liking through matrix-dependent interactions.

In summary, the integrated workflow combining untargeted LC/MS and GC/MS flavoromics with targeted GC/MS/MS chemical profiling of GC/O odorants enabled the identification of compounds that influence consumer preference among high-flavor-liking lager beer consumers. These findings demonstrate that both volatile and nonvolatile compounds, even at subthreshold concentrations or without inherent sensory attributes, can meaningfully influence consumer perception and overall liking. The compounds identified offer insights for flavor optimization and the development of lager products with enhanced sensory impact for fuller-flavored beer styles. However, while the current study advances the understanding of beer flavor chemistry, additional studies incorporating broader beer sample sets and consumers from different global regions could further refine the molecular drivers of beer liking and help define regional preferences. Moreover, because this study did not aim to identify all possible contributors to lager beer liking, the statistical criteria used to select the 14 predictive compounds may have excluded other relevant contributors.

## Supplementary Material



## Notes

GC-MS/MS MRM parameters for the quantitation of volatile predictive compounds in lager beer; mean liking scores, standard deviation, 95% confidence intervals, and Tukey’s HSD groupings for consumer study; LC-MS/MS MRM parameters for the quantitation of nonvolatile predictive compounds in lager beer; linear equations for standard addition quantitation curves of positively and negatively correlated compounds; and data will be available upon request (PDF)

## Abbreviations Used

GC-MS/MS-MRM: gas chromatography–tandem mass spectrometry with multiple reaction monitoring
GC/MS-O: gas chromatography–mass spectrometry–olfactometry
SAFE: solvent-assisted flavor evaporation
TF-SPME-SBSE: thin-film solid-phase microextraction–stir bar sorptive extraction
LC-MS/MS: liquid chromatography–tandem mass spectrometry
MRM: multiple reaction monitoring
LC/MS: liquid chromatography–mass spectrometry
LC/MS-QToF: liquid chromatography–mass spectrometry with quadrupole time-of-flight
PDMS: polydimethylsiloxane
PDMS/DVB: polydimethylsiloxane/divinylbenzene
GC/MS: gas chromatography–mass spectrometry
GC/MS/MS: gas chromatography–tandem mass spectrometry
OPLS: orthogonal partial least squares
PTFE: polytetrafluoroethylene
