# Supplementary material for: Volatile and Nonvolatile Contributors to Lager Beer Acceptability in High-Flavor-Liking Consumers
Source: J Agric Food Chem. 2026 Apr 28;74(18):14536–46. doi: 10.1021/acs.jafc.5c16579 (PMC13178071; doi:10.1021/acs.jafc.5c16579)
Supplement: Supplementary file 1 [file jf5c16579_si_001.pdf]

Supporting Information

Volatile and Nonvolatile Contributors to Lager Beer Acceptability in High-Flavor-Liking Consumers

Joel Borcherding, Megan Booth, Said Toro Uribe, Diana Paola Forero, Edisson Tello, Julie Peterson, and Devin G. Peterson*

Department of Food Science and Technology, Pelotonia Research Center, The Ohio State University, 2255 Kenny Rd, Columbus, OH 43210

*Corresponding author: Address: Department of Food Science and Technology, Pelotonia Research Center, The Ohio State University, 2255 Kenny Rd, Columbus, OH 43210.

Email: peterson.892@osu.edu

Table S1. GC-MS/MS Parameters for the Quantitation of Volatile Compounds using Multiple Reaction Monitoring (MRM) Mode.

**Compound**	**LRI**[a]		**Precursor Ion (*m/z*)**	**Product Ion (*m/z*)**		**Collision Energy (eV)**
	DB-Wax	DB-5		**Quantifier**	**Qualifier**	
Ethyl 2-methylpropanoate	955	700	116	88	73	2
Ethyl 2-methylbutanoate	1053	850	102	74	73	8
Ethyl 3-methylbutanoate	1067	854	88	61	60	5
2-Methyl-3-heptanone[b]	1174	939	128	86	71	5
Ethyl tiglate	1249	944	128	113	100	5
2-Acetylfuran	1518	893	110	95	67	10
Ethyl 3-methylthiopropionate	1587	1072	148	74	41	15
Geraniol	1849	1253	123	81	95	5
Dihydromaltol	1899	1094	128	43	57	10
2-Acetylpyrrole	2006	1072	109	94	66	10
4-Hydroxy-2,5-dimethyl-3(2H)-furanone	2045	1063	128	43	57	10

[a] Experimental linear retention index (LRI) on a DB-Wax column on two columns (DB-Wax and DB-5).
[b] Internal standard.

Table S2. Mean Liking Scores, Standard Deviation, 95% Confidence Intervals, and Tukey's HSD groupings (n=66).

Sample #	Mean Flavor Liking	Std. Deviation	Confidence Interval		Tukey HSD (α=0.05)
			Lower	Upper	
1	6.7	1.7	6.3	7.2	a
2	6.6	1.7	6.2	7.0	ab
3	6.4	1.9	6.0	6.9	abc
4	6.4	1.9	6.0	6.8	abc
5	6.1	1.8	5.6	6.5	abcd
6	5.9	1.9	5.5	6.3	abcd
7	5.8	1.6	5.3	6.2	abcde
8	5.8	2.3	5.3	6.2	abcde
9	5.8	1.8	5.3	6.2	abcde
10	5.5	1.9	5.1	6.0	bcde
11	5.5	1.9	5.1	5.9	bcde
12	5.5	2.1	5.1	5.9	bcde
13	5.5	1.9	5.0	5.9	cde
14	5.4	2.1	4.9	5.8	cdef
15	5.3	2.2	4.9	5.8	cdef
16	5.2	2.0	4.8	5.7	def
17	4.8	2.3	4.3	5.2	ef
18	4.3	2.1	3.9	4.7	f

Table S3. LC-MS/MS Parameters for the Quantitation of Nonvolatile Compounds using Multiple Reaction Monitoring (MRM) Mode.

Compound	Precursor Ion (*m/z*)	Product Ion (*m/z*)		Cone (V)	Collision Energy (eV)[a]
		Quantifier	Qualifier		
Pyroglutamyl leucine	243.10	86.08	132.05	22	18/12
Feruloylhydroxyagmatine	323.16	177.03	144.98	8	22/32
2-deoxyadenosine	252.03	136.00	119.03	18	18/42
p-coumaroyl hydroxyagmatine	293.16	147.01	119.05	30	26/36
N^1,N^{10}-diferuloylspermidine	498.29	177.02	144.97	48	36/48

[a] Collision energy for the quantifier ion/qualifier ion.

Table S4. Linear Equations for Standard Addition Quantitation Curves of Positively and Negatively Correlated Compounds

Compound	Correlation	Equation (Most Liked Beer)	Equation (Least Liked Beer)
Pyroglutamyl leucine	+	2.6593x + 0.0737	4.5099x + 0.1211
Feruloyl hydroxyagmatine	-	75.755x + 0.7168	23.445x + 0.2429
2-deoxyadenosine	-	43.338x + 2.0715	14.199x + 1.7127
p-coumaroyl hydroxyagmatine	-	93.993x + 1.95	26.748x + 0.5324
N1,N10-diferuloylspermidine	-	49.226x + 0.0173	14.71x + 0.0041
2-Acetylpyrrole	+	0.0846x + 58.8	0.0688x + 4.42
Ethyl 2-methylpropanoate	+	0.066x + 0.628	0.0457x + 0.044
Dihydromaltol	+	0.000211x + 1.19	0.000197x + 0.101
4-Hydroxy-2,5-dimethyl-3(2H)-furanone	+	0.000661x + 1.55	0.000373x + 0.122
2-Acetylfuran	+	0.0971x + 3.87	0.0731x + 0.458
Ethyl tiglate	+	0.0627x + 0.0717	0.0497x + 0.00331
Ethyl 3-methylbutanoate	+	0.195x + 0.795	0.143x + 0.0422
Geraniol	+	0.0458x + 8.9	0.0368x + 0.114
Ethyl 2-methylbutanoate	+	0.316x + 0.562	0.27x + 0.0352
Ethyl 3-methylthiopropionate	+	0.157x + 0.275	0.0976x + 0.0224
